# Increased Complement 3a Receptor is Associated with Behcet’s disease and Vogt-Koyanagi-Harada disease

**DOI:** 10.1038/s41598-017-15740-8

**Published:** 2017-11-14

**Authors:** Chaokui Wang, Shuang Cao, Dike Zhang, Hong Li, Aize Kijlstra, Peizeng Yang

**Affiliations:** 1 The First Affiliated Hospital of Chongqing Medical University, Chongqing Key Lab of Ophthalmology, Chongqing Eye Institute, Chongqing, P. R. China; 2 Xi’an No. 4 Hospital, Guangren Hospital of Xi’an Jiaotong University, Xi’an, P. R. China; 3 University Eye Clinic Maastricht, Maastricht, The Netherlands

## Abstract

Behcet’s disease (BD) and Vogt-Koyanagi-Harada disease (VKH) are systemic and recurrent autoimmune diseases associated with abnormal T cell immune response. Complement 3a receptor (C3aR) and complement 5a receptor (C5aR) have been reported to be involved in T cell mediated autoimmune disease. This study aimed to investigate the role of C3aR and C5aR in these two diseases. The C3aR expression in PBMCs was increased in patients with active BD (aBD) and active VKH (aVKH). No statistical difference was found concerning the expression of C5aR in PBMCs between patients with aBD or aVKH and normal controls. After the intraocular inflammation in BD and VKH patients was controlled, the C3aR expression returned back to normal levels. The serum from patients with aBD and aVKH significantly induced C3aR expression by PBMCs. C3a induced IL-6, IL-1β and TNF-α secretion, while inhibited the production of IL-10 by monocytes. Activation of C3aR in CD4^+^T cells could upregulate IL-17 production and inhibit IL-10 production, but had no detectable influence on IFN-γ production. Our data indicates that increased C3aR expression may lead to over activation of the Th17 cell response and may therefore contribute to the pathogenesis of BD and VKH disease.

## Introduction

Uveitis is the third leading cause of preventable vision loss worldwide^1^. There are two most common uveitis entities in China which are known as Behcet’s disease (BD) and Vogt- Koyanagi-Harada (VKH) disease^2^. BD is regarded as a chronic multisystemic autoinflammatory disorder, usually characterized by chronic and relapsing bilateral nongranulomatous, oral ulcers, genital ulcerations and skin lesions^3,4^. VKH disease is thought to be a multisystemic autoimmune disease, which usually appears as bilateral and diffuse granulomatous uveitis associated with alopecia, vitiligo, poliosis, central nervous system and auditory involvement^5,6^. It is well known that both Th17 and Th1 cell immune responses are involved in the pathogenesis of these two uveitis entities^7–10^. However, the exact immunological pathogenesis of BD and VKH remains unclear.

Complement 3a (C3a) and complement 5a (C5a) are produced after proteolytic cleavage of C3 and C5 in the process of complement activation. They are known as anaphylatoxins and have a short half-life ranging between 1 and 60 minutes. Their well-known effector function includes activation and chemotaxis of macrophages, granulocytes and mast cells^7^. Recent findings indicate that, in addition to its conventional roles in the innate immune system, C3a and C5a also regulate adaptive immune response by acting on antigen-presenting cells(APC) and T cells^7,8^. Previous studies demonstrated that locally produced C3a and C5a interacting with their receptor on APCs and T cells are involved in T cell differentiation^9^. Recent studies in both mice and humans have shown that C3a and C5a participated in regulating T cell responses by modulating dendritic cell (DC) function^10,11^. They reported that C3a and C5a could act directly on DCs, leading to an increased expression of co-stimulatory molecules and inflammatory cytokines, thereby affecting the T cell immune response. Animal studies revealed that the activation of C3a receptor(C3aR)/C5a receptor(C5aR) signaling on Treg cells inhibited the production and function of nTreg and iTreg cells. Blockade of the C3aR/C5aR signaling in nTreg cells led to more functional Treg cells, which could reduce the severity of autoimmune colitis and prolong the survival time of allogeneic skin graft^12,13^. Recent studies suggest that C3aR and C5aR participated in the development of experimental autoimmune uveitis(EAU), as evidenced by a marked reduction in the severity of inflammation and decreased pathogenic T cell responses in C3aR/C5aR-deficient mice^14^. These studies indicate that C3aR/C5aR signaling is important for the development of autoimmune response. However, it is not yet known whether C3aR/C5aR signaling is involved in human uveitis. In this study, we analyzed the level of C3aR and C5aR in BD and VKH disease, and found only C3aR was increased in active BD and VKH disease. The serum from active BD and VKH disease could induce the upregulation of C3aR in PBMCs, and the activation of C3aR could induce Th17-polarizing cytokines by monocytes and IL-17 production by CD4^+^T cells.

## Results

### Expression of C3aR and C5aR in PBMCs and monocytes from patients with aBD and aVKH

**Figure 1:**
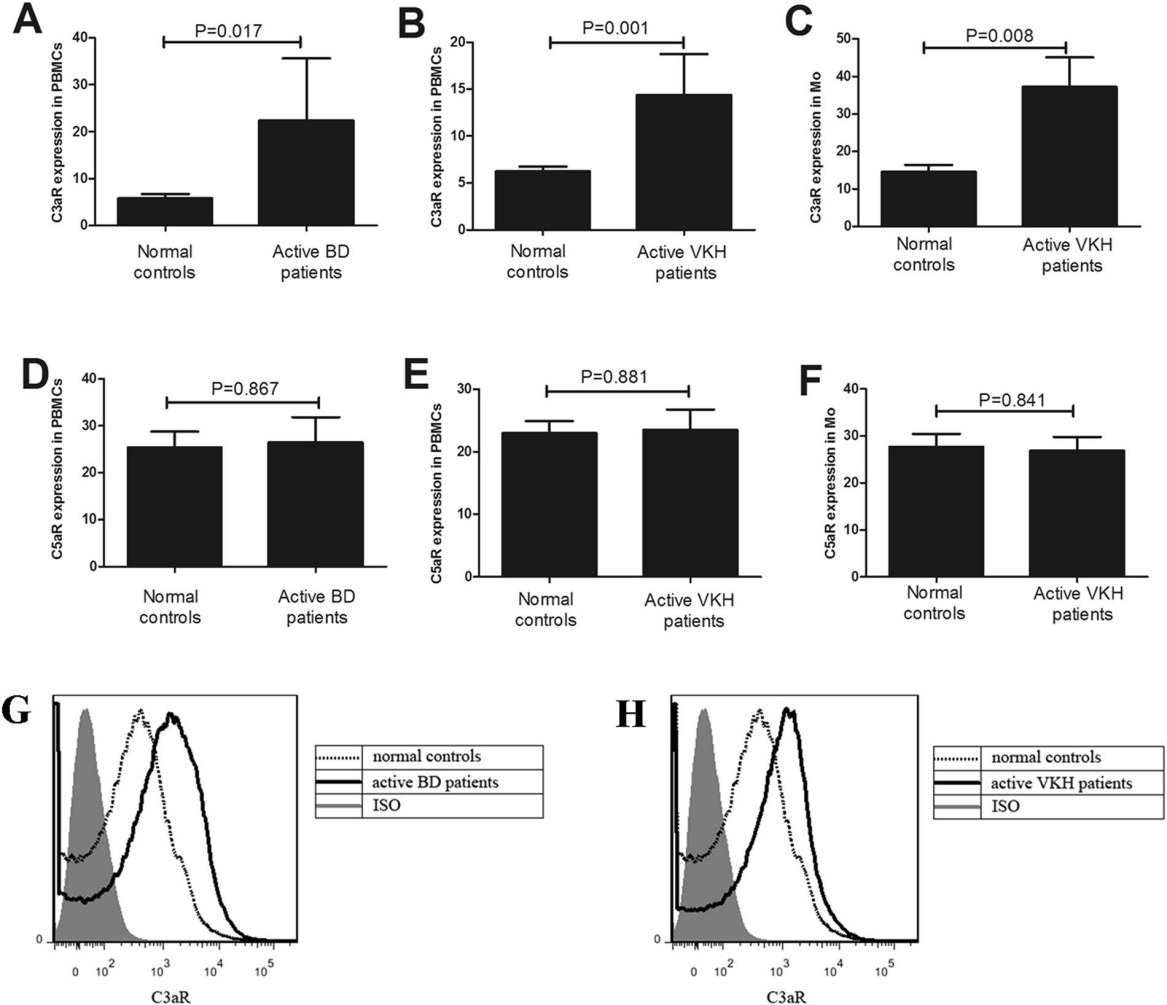
Expression of C3aR and C5aR in PBMCs and monocytes from patients with aBD and aVKH. (**A** and **D**) The expression of C3aR and C5aR was detected by FACS in PBMCs from patients with aBD (n = 5) and normal controls (n = 8). (**B**,**C** and **E**,**F**) The expression of of C3aR and C5aR was detected by FACS in PBMCs and monocytes from patients with aVKH (n = 18) and normal controls (n = 23). (**G**,**H**) Representative figures of the flow cytometric analyses of C3aR between aBD or aVKH and normal controls. Results are expressed as percentage difference compared with isotypic control. The Independent-sample test or Mann-Whitney U test was used to assess the difference of C3aR and C5aR level between patients with aBD or aVKH and normal controls. Data are expressed as mean ± s.e.m.

**Figure 2:**
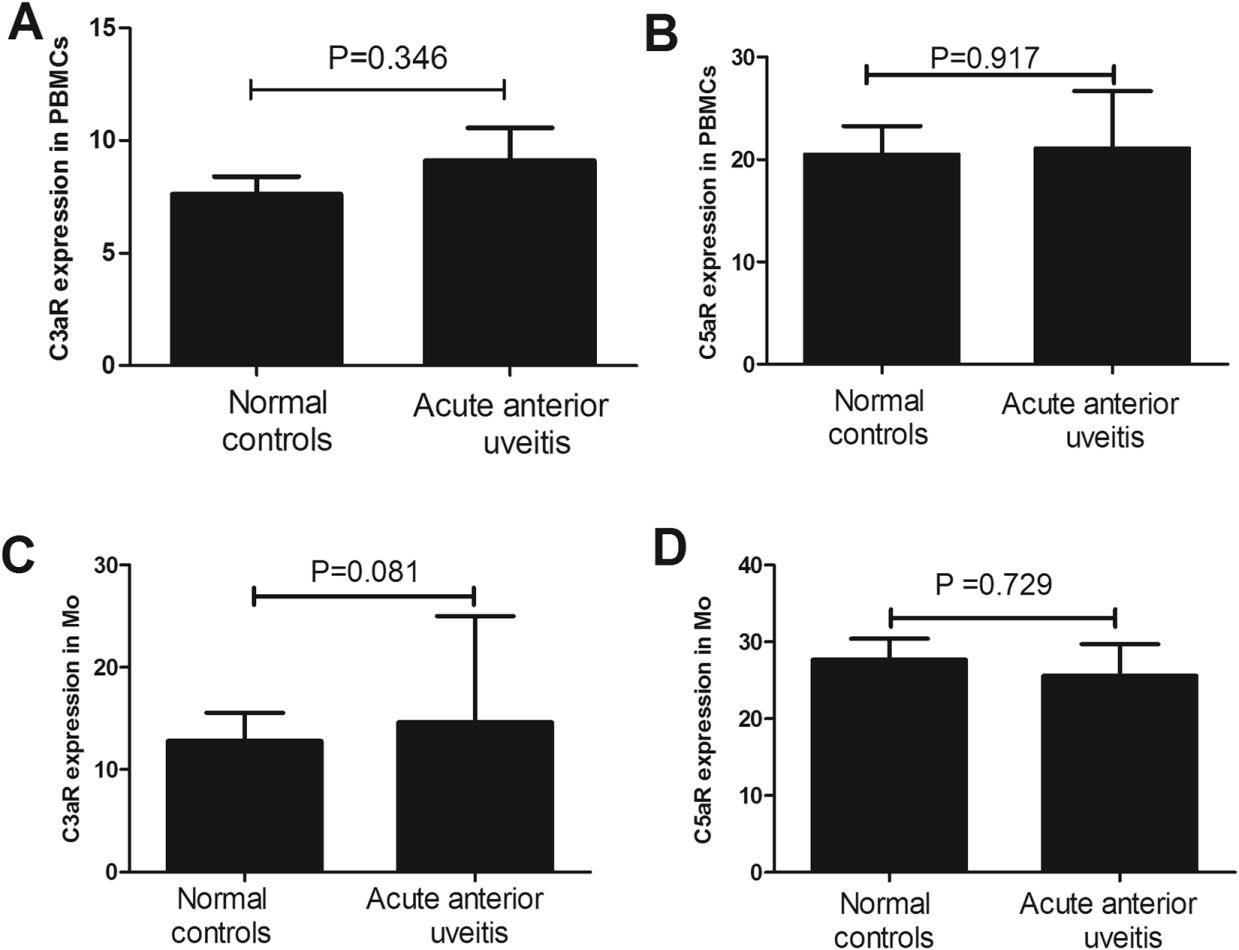
Expression of C3aR and C5aR in PBMCs and monocytes from AAU patients and normal controls. The expression of C3aR and C5aR was detected by FACS in PBMCs (**A**,**B**) and monocytes (**C**,**D**) from acute anterior uveitis (AAU) (n = 12) patients and normal controls (n = 17). The Independent-sample test or Mann-Whitney U test was used to compare the difference between AAU patients and normal controls. Data are expressed as mean ± s.e.m.

We first measured the levels of C3aR and C5aR in PBMCs and monocytes from patients with aBD or aVKH and normal controls using FACS. As shown in Fig. 1, the expression of C3aR was higher in PBMCs from patients with aBD (3.86 fold difference) or aVKH (2.38 fold difference) than that from normal controls (P = 0.017, P = 0.001, respectively, Fig. 1A,G,B,H).The expression of C3aR was also higher in CD14^+^monocytes from patients with aVKH than that from normal controls (P = 0.008, 2.56 fold difference, Fig. 1C). However, no significant difference was found concerning C5aR expression between patients with aVKH or aBD and normal controls in the two cell types investigated (Fig. 1D,E,F). Additionally, we examined the expression of C3aR and C5aR in idiopathic active acute uveitis (AAU) which served as a disease control group. We found no significant difference on the levels of C3aR and C5aR between AAU patients and normal controls (Fig. 2).

**Figure 3:**
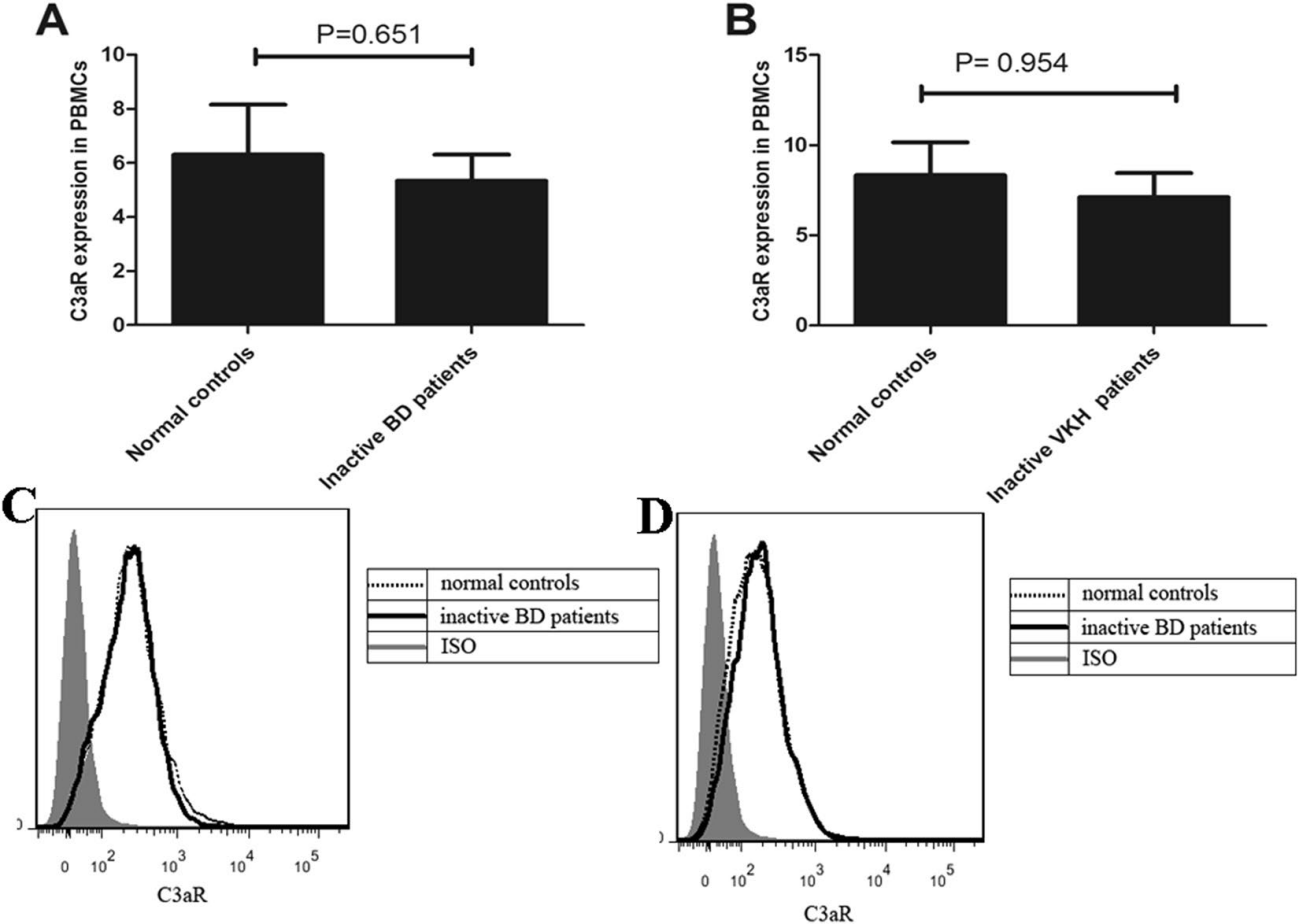
Expression of C3aR in PBMCs from inactive BD patients and inactive VKH patients. (**A** and **C**) The expression of C3aR was detected by FACS in PBMCs from inactive BD patients (n = 8) and normal controls (n = 8). (**B** and **D**). The expression of C3aR was detected by FACS in PBMCs from inactive VKH patients (n = 8) and normal controls (n = 8).The Independent-sample test or Mann-Whitney U test was used to compare the difference between patients with inactive BD or inactive VKH and normal controls. Data are expressed as mean ± s.e.m.

Experiments were also designed to examine whether C3aR expression was affected after successful treatment of patients with cyclosporin and steroids. PBMCs from inactive BD patients and inactive VKH patients were used to detect the expression of C3aR using flow cytometry. The results showed that there was no significant difference on the expression of C3aR between inactive BD patients (Fig. 3A,C) or inactive VKH patients (Fig. 3B,D) and normal controls.

### Serum levels of C3a and C5a in patients with aBD and aVKH

**Figure 4:**
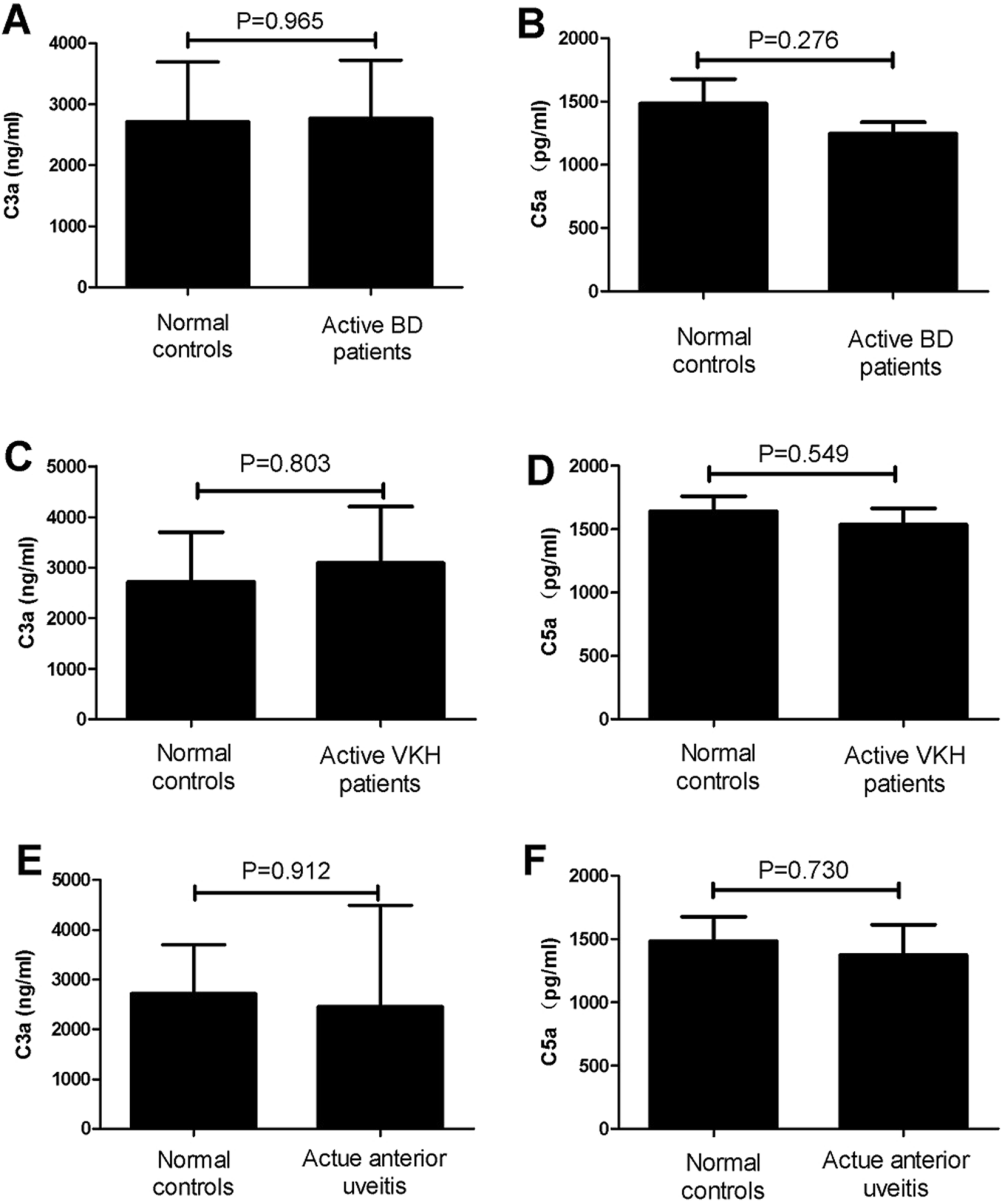
Serum levels of C3a and C5a in patients with aBD and aVKH. C3a and C5a levels in serum from patients with aBD (n = 16), aVKH (n = 13), AAU (n = 7) and normal controls (n = 13) were assessed by ELISA. The Independent-sample test or Mann-Whitney U test was used to compare the difference between patients and normal controls. Data are expressed as mean ± s.e.m.

The aforementioned experiment showed that there was an increased expression of C3aR, but not C5aR, in patients with aBD and aVKH. As C3a and C5a can induce their receptor expression respectively, a further study was performed to investigate C3a and C5a serum levels in patients with aBD and aVKH. The results showed that there was no significant difference concerning the C3a and C5a serum levels between patients with aBD or aVKH and normal controls. Additionally, we also detected the C3a and C5a serum levels in AAU patients. Similar to the findings in BD and VKH patients, no significant difference was found concerning C3a and C5a serum levels between AAU patients and normal controls (Fig. 4).

### Effect of serum from patients with aBD or aVKH on C3aR and C5aR expression in PBMCs

**Figure 5:**
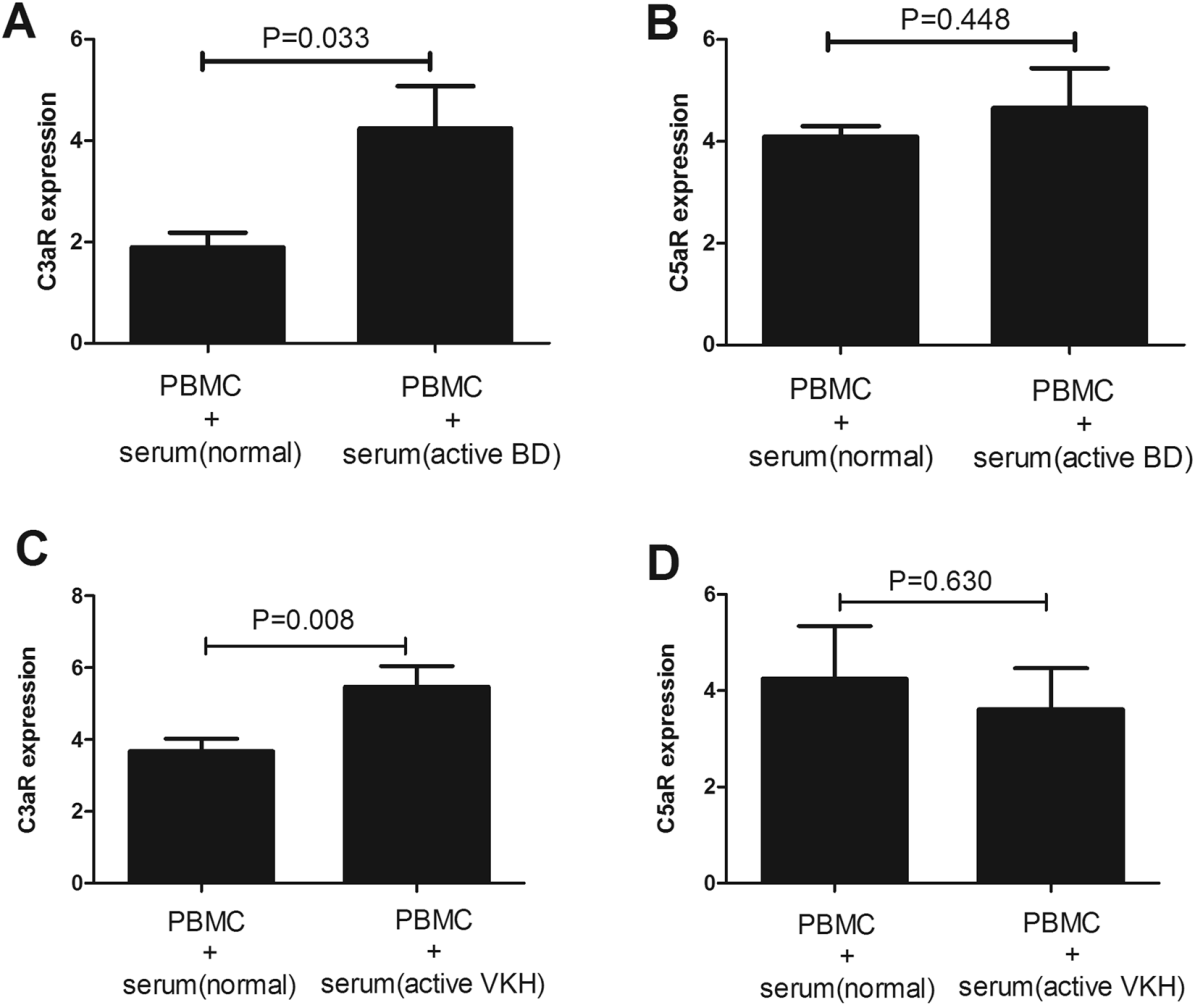
Effect of serum from patients with aBD or aVKH on C3aR and C5aR expression in PBMCs. (**A**,**B**) PBMCs were cultured in RPMI 1640 supplemented with 10% serum that from patients with aBD (n = 7) and normal controls (n = 11) in the presence of anti-CD3/CD28 for 3 days. (**C**,**D**) PBMCs were cultured in RPMI 1640 supplemented with 10% serum that from patients with aVKH patients (n = 13) and normal controls (n = 18) in the presence of anti-CD3/CD28 for 3 days. The expression of C3aR and C5aR in the cultured PBMCs was detected by FACS. The Independent-sample test or Mann-Whitney U test was used to compare the difference between patients with aBD or aVKH and normal controls. Data are expressed as mean ± s.e.m.

We subsequently investigated the effect of serum from VKH and BD patients on C3aR expression in PBMCs under conditions mimicking antigen stimulation. After culturing PBMCs with serum from patients with aBD, VKH or normal controls for 3 days, we analyzed the levels of C3aR and C5aR in the PBMCs using FACS. The results showed that C3aR expression was markedly higher in the PBMCs cultured with serum from patients with aBD (2.24 fold difference) or aVKH (1.49 fold difference) than that with serum from normal controls (P = 0.033, P = 0.008, respectively, 5 A and 5 C). No observable difference was found concerning C5aR expression in PBMCs cultured with serum from patients with aBD or aVKH and normal controls (Fig. 5B,D).

### Effect of C3aR activation on inflammatory cytokine production by CD14^+^ monocytes

**Figure 6:**
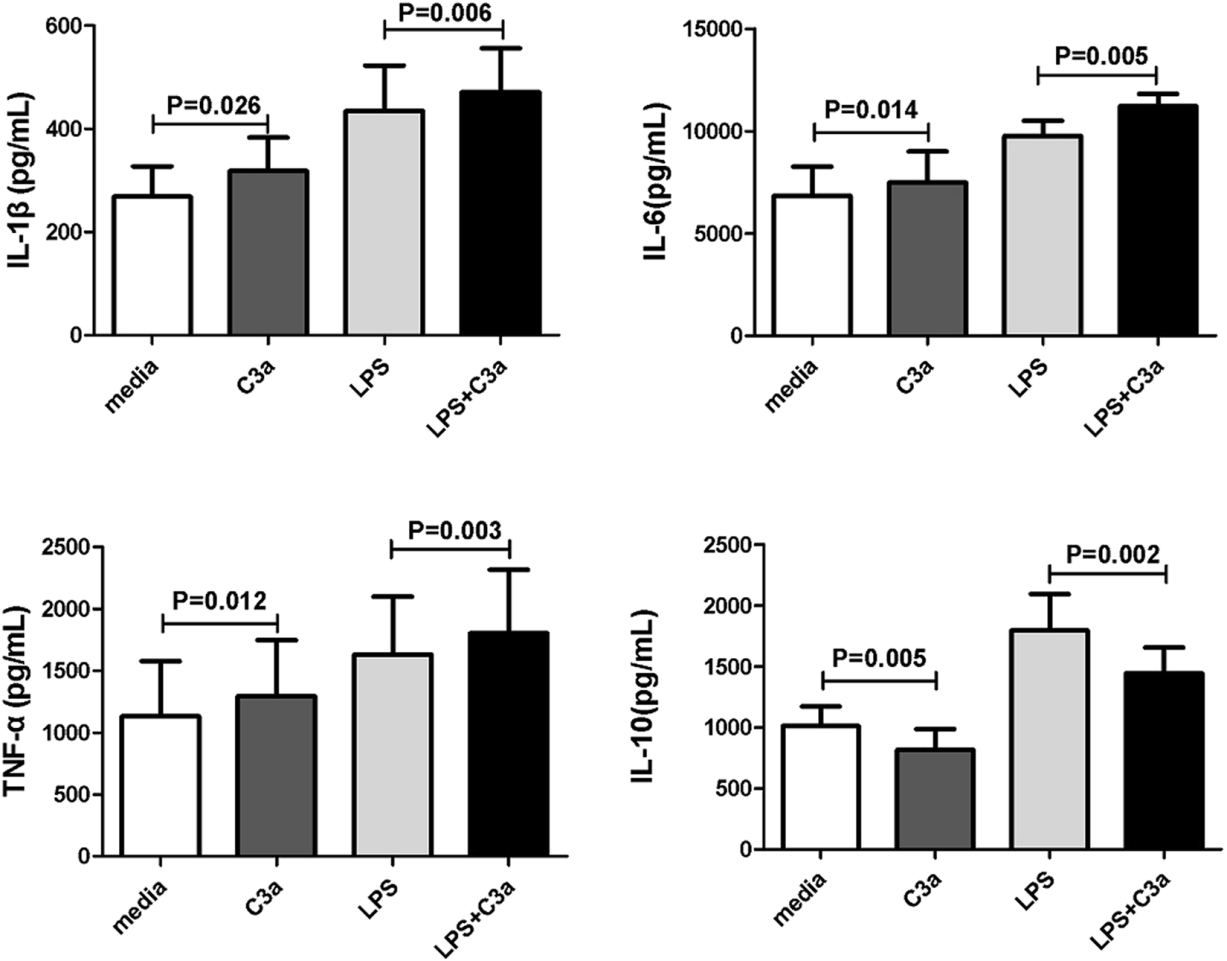
Effect of C3aR activation on inflammatory cytokine production by CD14^+^ monocytes. IL-1β, IL-6, TNF-α and IL-10 levels in the supernatants of monocytes stimulated with or without LPS (100 ng/mL) in the presence or absence of C3a (20 nM) for 3 days were measured by ELISA. Paired-samples t-test or Wilcoxon test for related samples was used for statistical analysis. All of the data are representative of five independent experiments with cells from eight donors and are expressed as mean ± s.e.m.

The aforementioned experiments suggest that an increased C3aR expression correlates with the activity of BD and VKH disease. We next studied the role of C3aR activation on the inflammatory cytokine production by monocytes. Monocytes were cultured in the presence or absence of C3a together with or without LPS for 72 h, and the cytokine levels of IL-1β, TNF-α, IL-10 and IL-6 in the supernatants were detected by ELISA. We found that C3a induced higher production of IL-1β (P = 0.006, P = 0.026), IL-6 (P = 0.005, P = 0.014) and TNF-α (P = 0.003, P = 0.012) by monocytes no matter with or without LPS stimulation. We also found that C3a significantly inhibited IL-10 production (P = 0.002, P = 0.005) by monocytes no matter with or without LPS stimulation (Fig. 6).

### Effect of C3aR activation on the inflammatory cytokine production by CD4^+^T cells

**Figure 7:**
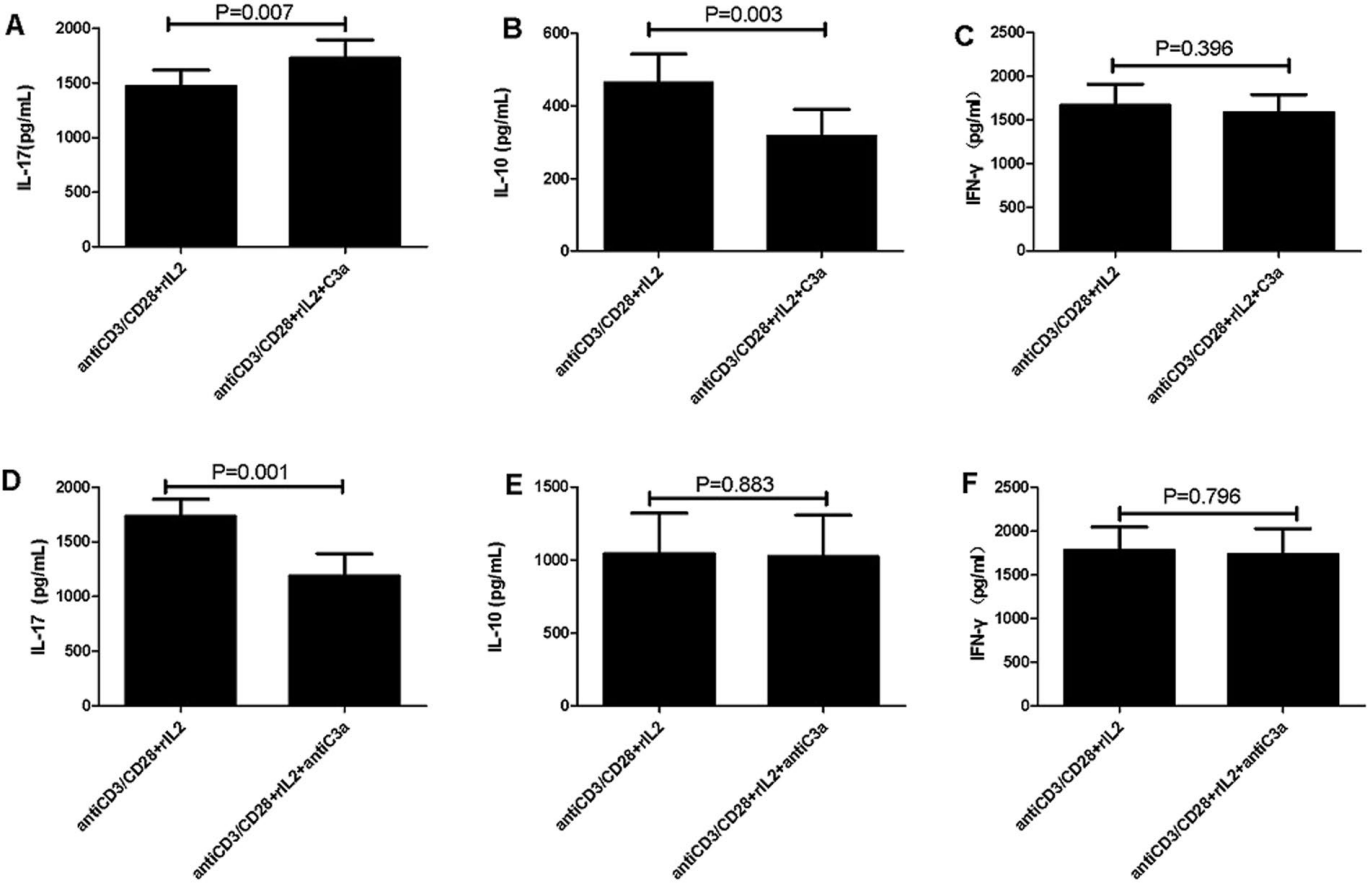
Effect of C3aR activation on inflammatory cytokine production by CD4^+^T cells. CD4^+^T cells were stimulated with anti-CD3/CD28 (0.5 μg/mL) in the presence or absence of (**A**–**C**) recombinant human C3a (50 ng/mL) or (**D**–**F**) purified anti-human C3a (10 μg/mL) for 5 days, IL-17, IL-10 and IFN-γproduction in the cell culture supernatants was determined by ELISA. Paired-samples t-test or Wilcoxon test for related sampleswas used for statistical analysis. All of the data are representative of five independent experiments with cells from thirteen donors and are expressed as mean ± s.e.m.

The previous study suggested that the C3aR was expressed on T cells, especially activated T cells^14^. We next investigated the role of C3aR activation in T cells. Sorted CD4^+^T cells were cultured in the presence or absence of C3a with anti-CD3/CD28 and IL-2 for 3 days, whereafter the cytokine levels of IL-10, IL-17 and IFN-γ in the supernatants were measured. We found that C3a significantly induced IL-17 production (P = 0.007), but inhibited IL-10 production (P = 0.003) by CD4^+^T cells. C3a did not have a detectable influence on IFN-γ production. Then we conducted a further study to investigate the effect of the blockade of C3aR signaling on T cells. We found that anti-C3a significantly inhibited IL-17 production (P = 0.001) by CD4^+^T cells, but had no influence on IL-10 or IFN-γ production (Fig. 7).

## Discussion

Our study showed that patients with aBD or aVKH had an increased expression of C3aR, but not C5aR, in their PBMCs compared to healthy controls. The serum from patients with aBD and aVKH could induce C3aR expression, but did not have a detectable influence on C5aR expression in PBMCs. *In vitro* experiments showed that C3a induced the production of IL-6, IL-1β, TNF-α, and inhibited IL-10 production by monocytes. Furthermore, we found that C3a may induce IL-17 production, while it inhibited IL-10 production by CD4^+^T cells. These data aforementioned collectively indicate that the increased C3aR signaling may contribute to the pathogenesis of BD and VKH disease.

The main function of C3aR/C5aR signaling includes activation and chemotaxis of mast cells, granulocytes and macrophages^7^. In addition to the conventional role of C3aR/C5aR signaling in the innate immune system, numerous studies have been performed to investigate the possible role of C3aR/C5aR signaling in the adaptive immune system. To our knowledge, the possible role of C3aR/C5aR signaling has not been investigated in uveitis. We provided here the first evidence concerning the role of C3aR/C5aR signaling in BD and VKH disease. Our study showed that an elevated C3aR expression was detected in patients with aBD and aVKH, although the C3aserum levels were similar between these patients and normal controls. The elevated C3aR expression was only seen in uveitis patients with BD and VKH disease, but could not be observed in AAU patients. The increased level of C3aR in the two diseases seems to suggest that C3aR signaling is correlated with the activity of both diseases. Interestingly, the clinical manifestation and immunopathology of the two uveitis entities are quite different. BD is characterized by a non-granulomatous uveitis and considered as an autoinflammatory disease, whereas VKH is a typical granulomatous uveitis caused by an autoimmune reaction against melanin associated antigens. The increased C3aR expression in active BD and active VKH patients, together with a similar effect of serum from both patient populations on C3aR expression, suggests that C3aR signaling is involved in both diseases, although the triggering factors may be different in these two diseases. Our findings are generally consistent with animal studies showing thatC3aR/C5aR double-KO mice exhibited less severe EAU and lower Th17 and Th1 cell response than control mice^14^.

Animal studies have reported that C5aR is involved in the adaptive immune response^12,13,15^. In our study, we could not detect obvious differences in the levels of C5aR and C5a between patients with BD or VKH or AAU and normal controls. Our findings are consistent with a previous study suggesting that there was no difference in disease severity or incidence between C5aR^−/−^ and wild type mice in the EAU model^16^. However, another study showed that treating EAU mice with an anti-C5mAb, which inhibits C5a release, reduced disease severity in EAU^15^. The discrepant findings with our results may be owing to the difference of immunological mechanism between clinical uveitis and animal models, and the discrepant finding between the EAU model studies may be due to the difference of housing conditions and analytical approach used to compare the disease severity.

Our findings revealed that there was an elevated expression of C3aR in patients with aBD and aVKH, but the exact reason why active BD and VKH patients have a higher expression of C3aR is not yet clear. We showed that serum from active BD or active VKH patients could induce a higher expression of C3aR compared with that from normal controls, indicating the existence of stimulatory factors in the serum from patients with aBD or aVKH. The identity of these circulating stimulatory factors is unknown and deserves further study. Whether these factors are associated with disease activity also deserves further investigation.

To investigate the possible role of C3aR signaling in the immune response, we firstly examined the effect of C3a on monocytes, and found that C3a could induce IL-6, TNF-α and IL-1β production, while inhibited IL-10 secretion, even in the absence of LPS stimulation. Our findings are in line with a recent study demonstrating that C3a induces IL-6 and TNF-α secretion by monocyte derived DCs^11^. Another recent study reported that C3aR activation alone did not influence the IL-1β production, whereby a combination of a C3aR agonist and LPS induced IL-1β secretion by monocytes^17^. The combination of LPS and C3aR agonist however did not modulate the secretion of IL-6. This latter finding is in disagreement with our results and may be due to differences in culture conditions, or due to the fact that a chemical C3aR agonist was used to activate C3aR. It has been reported that the differentiation of Th17 cell could be induced by TNF-α, IL-1β and IL-6, while being inhibited by IL-10^18^. Together, these data indicates that C3aR may contribute to the pathogenesis of BD and VKH disease possibly through promoting Th17 cell differentiation.

Since both Th17 and Th1 cells participate in the pathogenesis of BD and VKH^19^, we conducted a further study to determine the direct effect of C3a on Th17 and Th1 cell response. We found that C3a significantly induced IL-17 secretion whereas inhibited IL-10 secretion by CD4^+^T cells. Our findings are in accordance with previous reports revealing that C3aR signaling promotes the proliferation and differentiation of Th17 cells and inhibits the Treg cell function in animal studies^12–14^. However, we did not find a detectable effect on IFN-γ secretion by these cells. Earlier studies reported that C3a pre-treated DCs could induce IFN-γ production by T cells^11^. These discrepant findings are not yet completely clear. It may be possible that C3a could induce IFN-γ production by modulating DCs, but did not have a direct effect on IFN-γ by CD4^+^T cells.

In conclusion, the present findings revealed an increased C3aR expression in patients with aBD and aVKH. Furthermore, we showed that C3a could induce Th17-driving cytokine production by monocytes and directly promoted IL-17 secretion by CD4^+^T cells. The results mentioned above indicate that blocking C3aR signaling might be a strategy in treating BD and VKH disease, although more studies are needed to warrant this presumption.

## Materials and Methods

### Study subjects

In our study, the subjects were made up of 16 active BD (aBD) patients, 30 active VKH (aVKH) patients, 8 inactive BD patients, 8 inactive VKH patients and 14 idiopathic active anterior uveitis (AAU) patients. We also recruited 48 healthy volunteers as controls(HCs). Due to practical reasons we were not able to do all tests in all patients and controls. This explains why several analyses were done in a smaller group of subjects. We matched patients and controls for age and gender, although this was not always possible for the BD patient group due to an overrepresentation of males. The diagnosis of BD and VKH followed international criteria respectively^4,20,21^. None of the investigated patients used immunosuppressive agents or prednisone for at least two weeks before blood sampling. In our study, the patients with aBD were defined as BD patients with active uveitis, which are proved by dust keratic precipitates (KP) (100%), anterior chamber cells and flare (100%), vitreous cells(75%) and retinal vasculitis (100%).The active VKH patients were defined as VKH patients with active ocular inflammation evidenced by mutton fat KP (100%), anterior chamber cells and flare (100%), iris nodules(30%), and sunset glow fundus(60%). Written and informed consent was received from all patients and healthy controls. This study was performed on the basis of the basic principle of Helsinki declaration and approved by the Clinical Ethical Research Committee of the First Affiliated Hospital of Chongqing Medical University. All methods were performed in accordance with the relevant guidelines and regulations.

### Cell isolation and cell culture

We purified the peripheral blood mononuclear cells (PBMCs) from patients and normal controls by Ficoll-Hypaque density-gradient centrifugation. CD4^+^T cells and CD14^+^monocytes were separated from PBMCs by magnetic microbeads (both with a purity >90%, Miltenyi Biotec, Bergisch-Gladbach, Germany). In order to study the effect of serum from BD and VKH patients on the levels of C3aR and C5aR during antigen presentation, PBMCs (1 × 10^6^/mL) from normal controls were cultured in RPMI 1640 with 10% serum from patients with aBD or aVKH and normal controls plus anti-CD3/CD28 (0.5 μg/mL, eBioscience, San Diego, CA) for 3 days. The anti-CD3/CD28 cocktail was used to mimic antigen presentation. the levels of C3aR and C5aR were then analyzed by FACS. To determine the effect of C3aR activation on CD14^+^ monocytes, CD14^+^ monocytes (1 × 10^6^/mL) were cultured with or without LPS (100 ng/mL, Sigma-Aldrich) in the presence or absence of C3a (20 nM, R&D Systems) for 3 days, then the cytokine levels of IL-6, IL-1β, IL-10 and TNF-α in the collected supernatants were detected by ELISA. To investigate the influence of C3aR activation on theT cell response, CD4^+^T cells were stimulated with recombinant human C3a (50 ng/mL, R&D Systems) or purified anti-human C3a (10 μg/mL, Biolegend, CA, USA) plus anti-CD3/CD28 (0.5 μg/mL) for 5 days, then the cytokine levels of IL-17, IL-10 and IFN-γ in the supernatants were measured using ELISA.

### Flow cytometry

To study C3aR and C5aR expression in PBMCs and monocytes, the isolated PBMCs were washed and incubated with the following antibodies or isotype control antibodies in staining buffer at 4 °C for 30 min: anti-human C3aR-PE(Biolegend, CA, USA), anti-human CD88 (C5aR) - PerCP/Cy5.5 (Biolegend, CA, USA) and anti-human CD14-FITC (eBioscience, San Diego, CA).The samples were analyzed on a FACS Aria cytometer (BD Biosciences). The results were presented as [mean fluorescence intensity (MFI) of C3aR/C5aR – MFI of IC]/MFI of IC × 100% as described previously^22,23^.

### ELISA

Human Duoset ELISA kits (R&D System) were used to measure the cytokine levels of IL-1β, IL-10, IL-6, IL-17, TNF-α and IFN-γ in the culture supernatants of monocytes or CD4^+^T cells and the levels of C5a in serum. Human C3a platinum ELISA kit was used to detect the C3a level in patient and control serum (eBioscience, San Diego, CA).

### Statistics

Independent-sample test or Mann-Whitney test for unpaired samples and Wilcoxon test or paired-sample t test for paried samples were used to analyze the experimental data using SPSS13.0 software. P value less than 0.05 was regarded as significant.
